# The ethical challenges in pharmacy practice in community Pharmacies: A qualitative study

**DOI:** 10.1016/j.jsps.2021.11.003

**Published:** 2021-11-11

**Authors:** Rasool Esmalipour, Bagher Larijani, Neda Mehrdad, Abbas Ebadi, Pooneh Salari

**Affiliations:** a Medical Ethics and History of Medicine Research Center, and School of Medicine, Tehran University of Medical Sciences, Tehran, Iran; b Endocrinology and Metabolism Research Center, Endocrinology and Metabolism Clinical Sciences Institute, Tehran University of Medical Sciences, Tehran, Iran; c Diabetes Research Center, Endocrinology and Metabolism Clinical Sciences Institute, Tehran University of Medical Sciences, Tehran, Iran; d Nursing Care Research Center, Iran University of Medical Sciences, Tehran, Iran; e Behavioral Sciences Research Center, Life Style Institute, And Nursing Faculty, Baqiyatallah University of Medical Sciences, Tehran, Iran; f Medical Ethics and History of Medicine Research Center, Tehran University of Medical Sciences, Tehran, Iran

**Keywords:** Pharmacy ethics, Professionalism, Ethical challenges, Pharmaceutical care, Community pharmacy, Pharmacy practice

## Abstract

**Background:**

Pharmacists as a group of health care professionals, face different types of ethical challenges in their everyday routine that may impede pharmaceutical care.

**Objectives:**

In this study, we aimed at evaluation and recognition of the ethical challenges of pharmacy practice in community pharmacies.

**Methods:**

This exploratory study was conducted as a qualitative study consisting of open-ended in-depth interviews and focus group discussions followed by content analysis. The study participants were chosen from pharmacists with PharmD degree who had at least 4 years work experience and were the founders or technical managers of community pharmacies, either as governmental or private. Interviews continued until data saturation and transcribed verbatim. The content analysis was done by Graneheim and Lundman method. The codes were generated, and categorized. After assessment and final modifications, the results of the study were discussed and confirmed in a focus group discussion conducted by 7 experts who teach medical ethics and/or pharmacy ethics.

**Results:**

Overall, 40 pharmacists were interviewed (mean age 46 ± 11.3 years). The extracted ethical challenges of pharmacy practice were categorized into 3 main themes, 11 subthemes and 102 codes. The themes were achieved as challenges related to professionalism and professional practice, challenges related to professional communications and challenges related to regulations and policies.

**Conclusion:**

Taken together, it seems that most of the challenges of pharmacy practice are related to professionalism and professional commitment; however, the regulations and policies provide serious obstacles for pharmacy practice and pharmaceutical care. More efforts towards teaching professionalism and modification of regulations and policies are recommended.

## 1 Introduction

The pharmacy profession plays an important role in every health system (Mazhar et al., 2017). Historically, the role of pharmacists has changed over time in parallel with the advancements in knowledge and technology from drug dispensing to providing pharmaceutical care. This advancement presents new definitions of philosophy and standards in pharmacy practice (Salari and Abdollahi, 2017) by emphasizing pharmacists' professional responsibilities toward patients health. The American Pharmacists Association (APhA) (American Pharmacists Association, 2021)stated that “Pharmacists are healthcare professionals who assist individuals in making the best use of medications” (American Pharmacists Association, Code of Ethics for Pharmacists). According to the American Society for Health System Pharmacists (ASHP), pharmaceutical care is defined as “the responsible provision of medication-related care for the purpose of achieving definite outcomes that improve a patient's quality of life” (ASHP Statement on Pharmaceutical Care) (Statement, 2021).

The American Board of Internal Medicine (ABIM) developed fundamental principles of professionalism that have been adapted by Hammer for pharmacists (Chisholm-Burns et al., 2017). In addition, the American Association of Colleges of Pharmacy (AACP) and Academy of Students of Pharmacy (ASP) introduced 10 pharmacists’ professional tenets emphasizing professionalism (Chisholm et al., 2006). Being a health professional necessitates respecting ethical principles in providing pharmaceutical care. The Ethics Code of the Royal Pharmaceutical Society of Great Britain accentuates the public and professional interests and introduces the key responsibilities of a pharmacist (Deans, 2005; Royal Pharmaceutical Society of Great Britain. The Code of Ethics of the Royal Pharmaceutical Society of Great Britain) (The Royal Pharmaceutical Society of Great Britain, 2021). The Code of Ethics for the National Pharmaceutical System of Iran stresses the ethical and professional principles consisting of eight tenets including respect for autonomy of the patient, beneficence, non-maleficence, justice, empathy, excellence, integrity, and collaboration (Ministry of Health and Medical Education. The Code of Ethics for the National Pharmaceutical System of Iran) (Ministry of Health and Medical Education, 2021).

Respecting professionalism have a major impact on quality of health care services, public understanding of the profession, and trust in the profession which promotes pharmacist-patient relationship and patients’ accomplishment (Eukel et al., 2018) as well as patient’s adherence and compliance (Holwerda et al., 2013). Having that in mind, professionalism in pharmacy practice attributes to providing pharmaceutical care; however, it seems that pharmacists are not successful in achieving this goal.

Pharmacists are facing different kinds of challenges in pharmacy practice to provide pharmaceutical care including ethical, economic, clinical, and legal, that are as the main obstacles in health care provision. Because of this, there is a significant gap between the standard of pharmaceutical care and current pharmaceutical services (Javadi et al., 2018).

Upgrading quality of the pharmaceutical care necessitates determination of the ethical challenges, their origin, and finding the way into their resolution. The ethical challenges differ between cultures, so, the approach toward them may be different between countries.

In our country, a few studies have been performed to determine pharmacists’ ethical challenges; however those studies were conducted in one geographical location with less focusing on professional dimensions of ethical challenges (Javadi et al., 2018, Iranmanesh et al., 2020, Imaz et al., 2018, Reisnejadian et al., 2016, Salari et al., 2011); so, it seems that their findings are not conclusive. To have a more comprehensive exploration, this study was performed in the community pharmacies with two different types of management systems including governmental and private in two different geographical locations, Tehran and Tabriz (two large cities of Iran) with different cultures to explore the challenges of professional ethics to have a better perception of those challenges for further intervention into the modification of pharmacy practice by focusing on professional dimensions of the ethical challenges and pharmacists’ experiences.

## 2 Methods

### 2.1 Qualitative approach

This exploratory study was conducted from December 2019 to January 2021 through separate face-to-face interviews with pharmacists practicing in community pharmacies in Tehran and Tabriz and an online focus group discussion (FGD).

### 2.2 Researcher characteristics and reflexivity

Two out of 6 members of the research team were female. At the time of and during the study, RE was PharmD and Ph.D. candidate in Medical Ethics. BL, PS, and NM were academic members of a research institute. AE was an academic member of the school of medicine at the local university. All researchers were involved in all phases of the study except for conducting interviews which were performed by RE. The research team aimed at exploring the challenges of professional ethics to have a better perception of those challenges for further intervention into modification of pharmacy practice. The final codes will be used to design and validate a questionnaire to elucidate the pharmacists' attitude toward professional challenges from an ethical point of view. This may help to find priorities for the corrective approach.

### 2.3 Context and sampling strategy

The study participants who enrolled in the interview were pharmacists as the founders or technical managers of community pharmacies. The eligible participants were: i) having a PharmD degree, ii) being active in pharmacy practice as a technical manager or founder of the pharmacy, iii) and having at least 4 years of work experience. The study participants were chosen using the purposive sampling method based on practice settings from private and governmental community pharmacies because it is assumed that the ethical challenges of these two settings are different. The governmental pharmacies are community pharmacies affiliated to the school of pharmacies or the Red Crescent Society of the Islamic Republic of Iran. Because, governmental pharmacies are economically related to government and the economical issues and financial profit is not a concern for pharmacists while in private community pharmacises financial profit of the pharmacy is one of the main concerning issues. Customers of both types of pharmacies are the same but the payment system is completely different and the final monthly income of the private pharmacies may have negative impact on the remuneration of the pharmacists. To achieve a maximum variation of views, the community pharmacies of different parts of two cities, Tehran and Tabriz with different socio-cultural backgrounds were chosen.

### 2.4 Ethical issues pertaining to human subjects

Before enrollment, an invitation letter was emailed to each participant to explain the purpose of the study, the method of data collection, and to assure confidentiality and voluntary participation. Informed consent was obtained at the beginning of the interviews verbally. Each interview was conducted in a quiet and private environment. The study was approved by the research ethics committee of the School of Medicine, Tehran University of Medical Sciences, Tehran, Iran (IR.TUMS.MEDICINE.REC.1397.359).

### 2.5 Data collection instrument

**Fig. 1:**
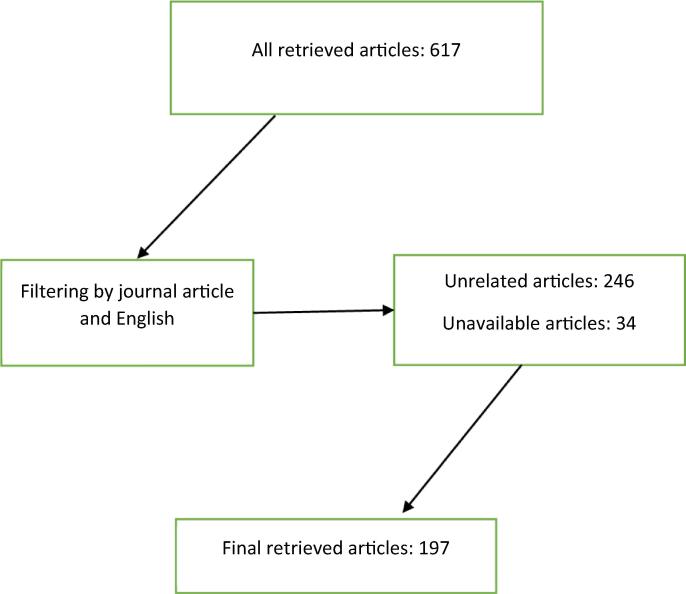
The article searches strategy and flowchart.

The first author (RE) searched the related literature with keywords including pharmacists, community pharmacy, professionalism, responsibility, pharmacy practice, and ethics using PubMed, Scopus, Cochrane and ProQuest databases. At first, 617 documents were retrieved; after filteration by different terms including journal articles only, and English language, and evaluation of the abstract to assess its compatibility, 197 were considered for content analysis. More details about search strategy are presented in Fig. 1. At this stage, different dimensions of pharmacy practice and pharmaceutical care were achieved that could be challenging from ethical and professional points of view. Considering those dimensions, the research team brainstormed seven open-ended questions as the interview framework that was finalized after reviews and corrections. This framework was used to persuade the participants to state their opinions about the ethical challenges they are facing in everyday routine. Table 1 provides a summary of the content of these questions.

**Table 1:** Summary of the content of the interview questions.

Interview topics
Having the experiences of professional challenges and facing dilemmas (describe and explain)
Having challenge in patient consultation
The experience in relationship with colleagues, physicians and other health care professionals
Having financial problems
Having challenge in drug dispensing according to quality of medication
The experience in relationship with drug distribution companies
Facing dilemma in choosing ethical versus legal approach

### 2.6 Data collection methods

Semi-structured interviews and an online FGD were performed for data collection and confirmation.

The average interview duration was 60 min. The interviews were extended until data saturation and obtaining no new information. The interviews were recorded by audio recorder and notes were taken during interviews and finally transcribed verbatim. Data saturation has been reached after 40 interviews, but 2 more interviews were conducted to ensure saturation.

After final modifications of the results of the interviews, to know and sift possible gaps in data and relevant results, an online FGD was conducted for 120 min and recorded. Because of the COVID-19 pandemic, the focus group discussion was online. The generated themes, subthemes, and codes were discussed for further validation and confirmation.

### 2.7 Units of the study

Overall, 40 participants aged 46 years (SD 11.3) who had 3.12 years (SD 1.01) of work experience were enrolled in open-ended in-depth semi-structured interviews. Of 40 participants, 12 were technical managers of the community pharmacies, 15 were technical managers of the governmental pharmacies and 13 were founders of community pharmacies. More demographic details were presented in Table 2.

**Table 2:** Demographic data of the interviewees:

Interviewees (N: 40)	N	%
Age (year) (mean ± SD)	46.05 ± 11.323	
Technical managers of the community pharmacies	12	
Female	5	42
Male	7	58
Technical managers of the governmental pharmacies	15	
Female	8	54
Male	7	46
Community pharmacy founders	13	
Female	2	15
Male	11	85
Work experience		
5 yrs >	3	7.5
5–10 yrs	9	22.5
10–20 yrs	8	20
20 yrs <	20	50

The FGD was performed by seven experts who teach medical ethics and/or pharmacy ethics including a medical ethics specialist, a professor of pharmaceutics, two professors of clinical pharmacy, and three members of the research team.

### 2.8 Data processing, analysis, and confirmation

Data analysis was performed using a general deductive approach. After the first interview, the transcriptions were coded which continued until the fifth interview. The Graneheim and Lundman method was used for content analysis (Granheim and Lundman 2004). The MAXQDA software version 18.2.0 was used for the first round of content analysis and 126 codes were extracted categorized into 5 themes and 18 subthemes. Similar codes were merged and the themes were further refined. The primary draft of the codes, themes and subthemes were presented to the focus group. Some of the codes were considered as the reasons for ethical challenges and not related to the aims of the study; so, those codes were deleted. Finally, the focus group considered the second round of content analysis. The second round was conducted by two of the main researchers and evaluated, modified, and finalized by the rest of the research team. Finally, 3 themes, 11 subthemes, and 102 codes were obtained.

### 2.9 Techniques to enhance trustworthiness

Half of the transcripts were returned after the member check. The rest of the interviewees were reluctant to member check because of their busy schedules.

### 2.10 Reporting

This study is reported according to the “Standards for Reporting Qualitative Research (SRQR) guideline (Óbrien et al., 2014).

## 3 Results

Contrary to the first assumption of different ethical challenges in two different settings private and governmental pharmacies, we found similar results.

**Fig. 2:**
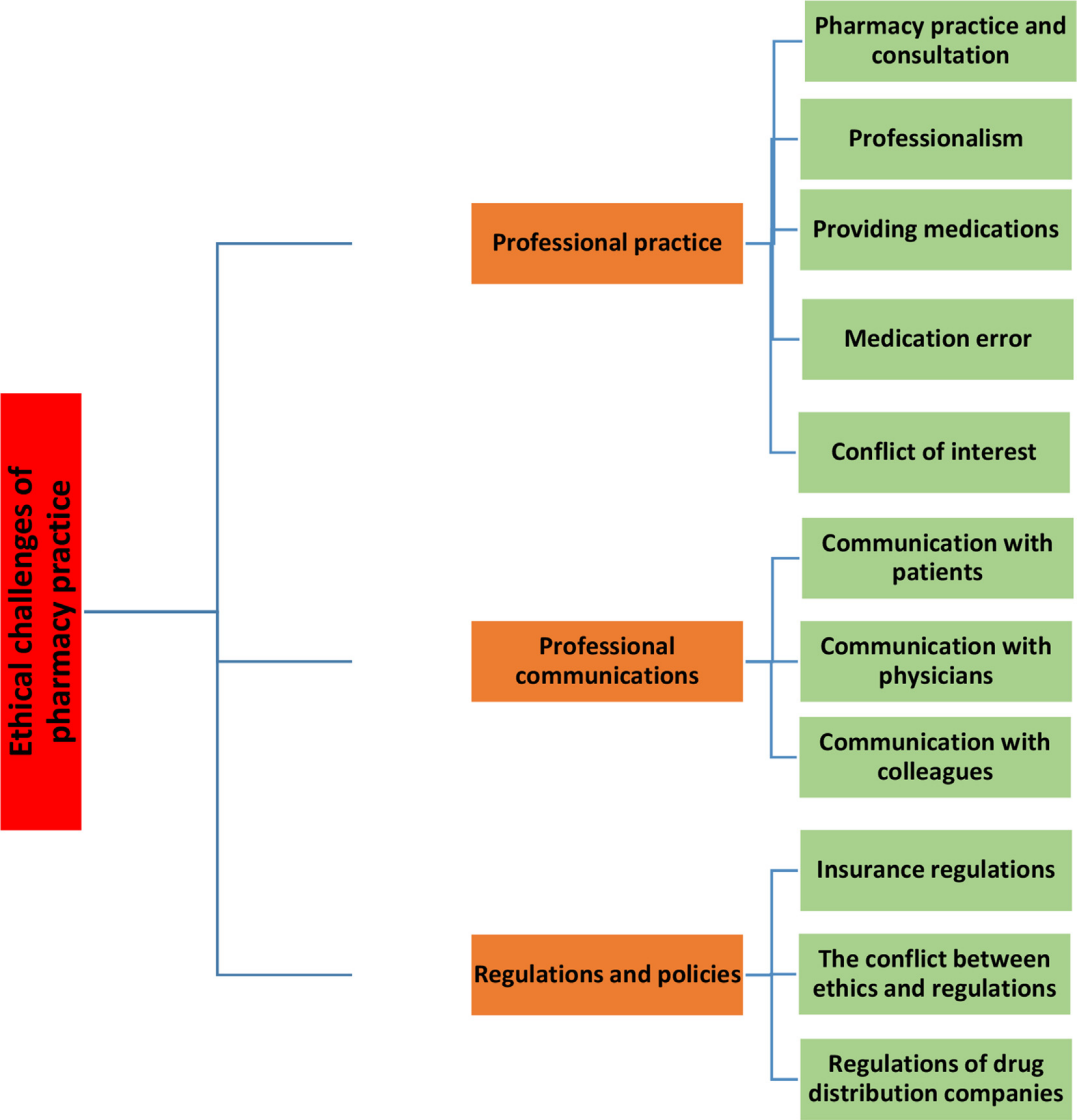
The obtained themes, Subthemes and Codes.

According to our findings, the ethical challenges of pharmacy practice are categorized into 3 major themes including challenges related to professionalism and professional practice, challenges related to professional communications, and challenges related to regulations and policies. The final themes are more explained as below. It could be noted that the themes and subthemes may have overlap; so, complete separation of them is not possible. They are presented in a concept map in Fig. 2.

### 3.1 Theme 1: Professionalism and professional practice

This theme consists of 5 subthemes including ethical challenges of pharmacy practice and consultation, respecting professionalism, providing medications, facing medication error, and conflict of interest. This theme was emphasized by participants who considered pharmacy practice and consultation as very important.

According to the interviewees, the ethical challenges originate from not having proper and enough medications called drug shortages, counterfeit medications, dealing with patients who wish to return medicines, and giving prescription drugs without physician’s order. Some participants stated:

*“…. Sometimes I have many ethical dilemmas in giving prescription drugs without physician’s order when I know that this medicine is not useful to the patient and the patient asks for it seriously”. [Participant No. 1]*

*“Sometimes I receive medication order in which the physician ordered or recommended counterfeit medicines. For examples, the physician has ordered nimodipine for cerebral ischemia. I know that nimodipine is not available but some pharmacists have imported it via informal pathways. May I provide this drug because the patient needs it? What should I do? “. [Participant No. 13]*

*“When patients request prescription medications without physician’s order, we face lots of challenges. For example, some patients who take antihypertensive or antihyperglycemic medications do not have access to their doctors; some others don’t have enough money to pay for doctor’s visit. Besides, some medications are not under the coverage of the patient’s insurance. In these situations, what are my responsibilities and duties? How we can provide service? Mostly, patients are forcing us to receive their medications”. [Participant No. 30]*

*“One of the challenges that affect pharmacy practice is poverty of the patient. Often, we meet patients who are not able to pay for medications especially the medications they take for their chronic diseases or cancer. This makes a big problem for us”. [Participant No. 16]*

As the professionalism points to the pharmacists responsibility in providing patients care, the subtheme of patients care could be considered as a hidden issue in this theme that could not be ignored.

The study participants had difficulties and faced ethical challenges because of medication errors. Accordingly, encountering medication errors was confusing for them. A participant stated:

*“The physician's order shows an incorrect drug dosage for example cefixime 400 mg BID. I know that this dosage is wrong. May I change the dose? Should I refer the patient to the physician? May I refer all orders with medication errors to the physician? It's not possible. If the physician denied and did not accept his mistake what should I do? Am I responsible to dispense the medication with the wrong dosage?” [Participant No. 13]*

### 3.2 Theme 2: Professional communications

Professional communications were the other theme of the ethical challenges. Communication skills are one of the most essential teachings for health professionals especially pharmacists; otherwise, they may encounter difficulties in pharmacy practice.

This theme consists of 3 subthemes including ethical challenges of communication with patients, communication with physicians, and communication with pharmacists. The participants stated:

*“There are many problems in the physician-pharmacist relationship. Communication skills do not teach in schools of medicine and pharmacy. Each of them may behave regardless of the other. They must be taught how to communicate with each other”. [Participant No. 28]*

*“Patient’s behavior should be modified. When they are in pharmacy, they must not hurry for receiving their medications. Some people think that they don't need consultation and guidance about their medications”. [Participant No. 36]*

### 3.3 Theme 3: Regulations and policies

This theme consists of 3 subthemes including ethical challenges related to regulations of health insurances, the conflict between ethical principles and regulations, and regulations of the drug distribution companies. Many of the participants stated that the most important challenges in the community pharmacies are related to regulations and policies which are not comprehensive and updated in which the pharmacists position in the health system are disregarded. Some of the regulations and policies goes back to 1953 when the “pharmaceutical care” was not a major concern. In this regard, some of the participants stated:

*“The medication distribution system is not fair and justified. They allocate some special medications for some selected community pharmacies in high amounts, but when we are asking for those drugs, they give us a limited amount. In such situations, I cannot provide enough medication to address the patient’s needs.” [Participant No. 12]*

*“Another problem in the community pharmacy is having medications beyond their expiry dates. We don't have clear regulation about expired medicine to dispose them; while, lots of patients ask us about drug disposal and we don't have a reasonable answer. Usually, the expired drugs are put in garbage or creek”. [Participant No. 16]*

*“…..In our country, the insurance reimbursement for medications is limited for 3 months and after that, the patient should take a doctor’s visit and pay again to be able to take his medications. This cycle is repeated every 3 months. [Participant No. 39]*

*“Some wholesalers have special offers that seduce us to buy more. For instance, buy …. Boxes of …. (drug name) and receive some gifts. I think these offers ruin our professional relationship. Furthermore, if we buy something more than our needs, we will try to sell it as soon as possible.” [Participant No. 16]*

## 4 Discussion

In this study the extracted ethical challenges of pharmacy practice in community pharmacies were categorized into 3 themes including challenges related to professionalism and professional practice, challenges related to professional communications, and challenges related to regulations and policies. At a glance, it seems that the first two themes are closely linked to each other and may have overlap; however, the first theme, mainly introduces the challenges related to providing pharmaceutical care to patients regardless of communications, while the second theme mostly emphasizes on the challenges arising from communication-an important and ignored part of each relationship. In fact, in this study pharmacy practice in its traditional way (drug dispensing) was not our concern; in contrast we mainly focused on the new approach toward pharmacy practice via providing pharmaceutical care.

### 4.1 Challenges related to professionalism and professional practice

Professionalism is defined as “the active demonstration of the traits of a professional” (Chisholm et al., 2006). In our study, the ethical challenges of professionalism were emphasized by all pharmacists as challenges of pharmacy practice and consultation, professional commitments, providing medications, medication error, and conflict of interest.

In line with our study, the study of Iranmanesh et al. shows different ethical challenges in community pharmacy including privacy and confidentiality, pharmacists’ awareness of their own professional commitment, considering patient’s interests, responsibility, quality of medication, and rational drug use (Iranmanesh et al., 2020). The financial problems of pharmacists, public unawareness about the pharmacists’ responsibility, insufficient teaching of professional ethics in the school of pharmacies, paternalism in the health system and giving gifts by companies were indicated as factors of creating the ethical challenges in pharmacy practice (Javadi et al., 2018). A study in Jordan indicated pharmacists’ ethical dilemma because of barriers such as lack of time, lack of ethical knowledge, non-expertise in ethical decision making, and not following the code of ethics (Al-Qudah et al., 2019). Pharmacy practice and consultation is the most important professional responsibility of pharmacists that results in the improvement of patients’ quality of life; without that pharmaceutical care is not achievable. Providing pharmaceutical care necessitates that pharmacists be the true ‘professionals,’ who take the responsibility of patient care to achieve optimal therapeutic outcomes (Chisholm-Burns et al., 2017).

Some of the challenges result from not being able to provide pharmaceutical care and consultation because of a wide variety of reasons including small workplace and chaos, the conflict between regulations and religious beliefs, patients request for medicine without a prescription, patients request for counterfeit medicine, shortage of medicine, facing with children’s request for medicine, bad news and truth-telling, confidentiality, unreliable quality of medications, and encountering irrational prescribing (Imaz et al., 2018). Another challenge that was achieved in this study, is patients unwilling to get consultation and pharmaceutical care that is unique and specific to our country and a major barrier in pharmaceutical care. This behavior seems to root in our perspective on health. In contrast, in a study in England, 92% of patients indicated that they are ‘very satisfied’ or ‘satisfied’ with their appointment with the pharmacist and 97% showed that they were ‘very comfortable’ or ‘comfortable’ discussing their medications with the pharmacist. Besides, 95% of patients were strongly agreed or agreed with the clinical recommendations of the pharmacist (Nabhani-Gebara et al., 2020).

Truth-telling is considered a duty of pharmacists by American Pharmacists Association (American Pharmacists Association. Code of Ethics for Pharmacists), but truth-telling is an ethical challenge in pharmacy practice because of a lack of education about how to properly communicate to different patients with different cultures (Salari et al. 2013), which is in line with our study results. In concordance with our findings, respecting patients’ confidentiality was confirmed as an ethical challenge in previous studies (Javadi et al., 2018).

Discrimination in pharmacy practice is an unethical behavior especially during drug shortage. Observing distributive justice is the ethical commitment of every health professional and access to medications is considered as a patient right in national and international guidelines. Accordingly, every patient should be served based on his needs and considering justice and fairness in drug supply and distribution (Salari et al., 2013). However, because of medication shortage, discrimination in pharmacy practice is specific to our country.

In addition, at the time of drug shortage, the pharmacists face patients request for counterfeit medications that deliberately and fraudulently mislabeled to source and/or identity. At least 10% of the medications available in the market are counterfeit medications and they are considered as a threat to patient safety in both developed and developing countries (Hosseini et al., 2011). Probably, pharmacists are interested in providing counterfeit medications because of the financial interest or helping the patients which is an ethical and professional dilemma. The quality of medicine was another ethical challenge for study participants because it affects effectiveness. All healthcare providers must be always benevolent to the patients while the low-quality medications can be ineffective or harm the patient.

The studies show medication errors by pharmacists increase mortality and morbidity (Ibrahim et al., 2010). Lack of a systematic approach toward medication error was the other finding of this study that diminishes patients’ confidence and increases health care costs. Detecting and preventing medication errors is the responsibility of pharmacists (Ibrahim et al., 2010); however, the patient has the right to know about medication error.

Community pharmacists should consider two conflicting dimensions in their work; the business and the professional dimensions (Sadek et al., 2016, Resnik et al., 2000). In pharmacy practice, conflicts of interests has two different forms including the conflict in communication with physicians (fee-splitting and self-referral) and conflict in communication with patients (Esmalipour and Parsa 2017). In agreement with our findings, the study of Raisnejadian et al. showed the conflicts of interests as one of the important ethical problems in community pharmacy (Raisnejadian et al., 2016).

### 4.2 Challenges related to the professional Communications

The ethical challenges of professional communications with patients, physicians and colleagues were the second theme of the pharmacists’ challenges. The study of Kruijtbosch et al stressed on the pharmacists contact with patients and health care professionals as the predominant moral dilemma which is complicated by other parties including regulators (Kruijtbosch et al., 2018).

Communication between pharmacist and patient affects patient satisfaction, medication use, and treatment outcome (Olsson et al., 2014). Also, effective pharmacist-patient communication reassures about safe medication use. In 2000, The World Health Organization introduced the “Seven Star Pharmacist” concept, which introduces the pharmacists as “communicators” (Nakayama et al., 2016). Therefore, pharmacists must be aware and knowledgeable about communication skills and adapt ethical principles in their professional behavior. The British Medical Association [BMA] (British Medical Association, 2012) strongly emphasizes the importance of all health professionals being properly trained to communicate in an honest and supportive manner (Brannan et al., 2012).

Other than patients, the pharmacist-physician relationship should be professional, scientific and logical. The study participants considered their relationship with physicians as one of the ethical challenges especially when they have a piece of advice about adverse drug reactions or drug interactions. Mostly, the physicians do not accept pharmacists’ advice while the studies show that pharmaceutical services can greatly reduce the total cost of care and the length of hospitalization as well as improving clinical outcomes (Mazhar et al., 2017, Hepler and Strand, 1990). This problem could originate from not being aware of the role of community pharmacists as the members of a multidisciplinary health care team.

Because of the shared responsibility between physicians and pharmacists in providing health care, knowledgeable pharmacists may be more sensitive to the physicians’ fault which could have a negative impact on their relationship (Salari and Abdollahi, 2017). Therefore, the pharmacists-physicians communication is a two-way relationship and both expect to behave respectfully (Mercer et al., 2020).

### 4.3 Challenges related to regulations and policies

The ethical challenges of regulations and policies were the last obtained theme in this study. When there is a conflict between ethical and legal responsibilities, pharmacists face ethical dilemmas. Mainly, the pharmaceutical system of Iran is under governmental control and specific regulations of the insurance companies. Our results show that in working with insurance and drug distribution companies, challenges originate from the conflict between regulations and ethical principles. Astbury et al. believe that policies, legislation and regulations, the structural and relational dimensions of working environment of pharmacists can cause moral distress (Astbury and Gallagher, 2020).

Sometimes pharmacists encounter conflict between legal considerations and professional ethics (Lowenthal, 1988). A study showed that there is no legal support for doing professional ethics in community pharmacy (Iranmanesh et al., 2020); while providing adequate support helps them accomplish their professional role toward the standard of care (Mercer et al., 2020).

The relationship of insurance companies with the pharmacist must be mutual; however, sometimes insurance companies do not carry out their commitments versus pharmacists and restrict pharmacist’s autonomy (Javadi et al., 2018). The enforcement of insurance companies for delivering generic drugs instead of brand names and frequent changes of their regulations not only diminish patients’ and pharmacists autonomy but also can cause distrust to pharmacists (Javadi et al., 2018).

According to our participants, unethical behavior of drug distribution companies such as selling unused medications to pharmacies, offering drug baskets, selling drugs with gifts, and unjustified drug distribution between community pharmacies create ethical challenges. Because of such problems, pharmacists sometimes ignore the quality of products that affects their professional behavior and decision-making (Volochinsky et al., 2018).

### 4.4 Strengths and limitations

This study elucidates similar ethical challenges of pharmacy practice in community pharmacies both in governmental and private systems. The main strength of this study was focusing on the professional dimensions of the ethical challenges. Also, the inclusion of two large cities with different cultures potentiates the findings. The high number of study participants, using a private environment for the interview, and the interviewees' comfort and ease during the interview were the other positive points of this study.

There are some limitations to this study. Lack of member check of half of the transcripts may weaken the credibility of data. Regarding the location of our study which was limited to our country, Iran, the generalizability and transferability of the findings are limited. Accordingly, some of the above-mentioned challenges are specific to our country such as patients' requests for counterfeit medicine and patients unwilling to take pharmacist consultation. The interviewer was a pharmacist which may affect the interviewees' response more desirable. Furthermore, there is the possibility that the interview questions bias the responses.

## 5 Conclusion

Three themes of ethical challenges in pharmacy practice in community pharmacies were discovered in this study including challenges of professionalism and pharmacy practice, challenges of professional communications and challenges of regulations and policies. It seems that we still stayed at the low level of the pharmacy practice and pharmaceutical care in community pharmacies.

To prosper pharmacy practice and to reach an acceptable level of pharmacists contribution in the health system, ethical challenges need to be overcome. Some of the challenges raise by external factors that can be modified by changing the educational model and teaching professionalism, and communication skills, and alteration of rules and regulations of the Food and Drug Organization of Iran toward more compatibility with law and ethical principles. However, the internal factors that are related to individual characteristics of the pharmacists as well as physicians and patients’ should not be ignored.

Furthermore, upgrading patient’s perspective on health will revive the pharmacy profession and helping in retrieving pharmacist’s motivation toward providing pharmacy practice and pharmaceutical care.

## CRediT authorship contribution statement

**Rasool Esmalipour:** Conceptualization, Methodology, Validation, Formal analysis, Investigation, Data curation, Writing – original draft, Project administration. **Bagher Larijani:** Conceptualization, Methodology, Project administration, Supervision, Writing – review & editing. **Neda Mehrdad:** Conceptualization, Methodology, Validation, Formal analysis, Data curation, Writing – review & editing. **Abbas Ebadi:** Conceptualization, Methodology, Validation, Formal analysis, Data curation, Writing – review & editing. **Pooneh Salari:** Conceptualization, Methodology, Validation, Formal analysis, Investigation, Data curation, Writing – original draft, Project administration, Supervision.

## Declaration of Competing Interest

The authors declare that they have no known competing financial interests or personal relationships that could have appeared to influence the work reported in this paper.
